# A Challenging Correlation between Tumor Cellularity and Somatic Variant Allele Fraction in Lung and Colorectal Cancers—Specimens of Low Tumor Percentage Should Be Analyzed with Caution

**DOI:** 10.3390/biom14020168

**Published:** 2024-01-31

**Authors:** Samaneh K. Zarabi, Lidong Zhai, Yu-Wei Cheng

**Affiliations:** 1 Department of Pathology, Stony Brook University Hospital, New York, NY 11794, USA; 2 Department of Laboratory Medicine, Robert J. Tomsich Pathology and Laboratory Medicine Institute, Cleveland Clinic, Cleveland, OH 44195, USA

**Keywords:** tumor percentage, tumor cellularity, somatic, variant allele fraction, correlation, sporadic cancer, NGS, NSCLC, CRC

## Abstract

**Background and aims:** The percentage of tumor cells (tumor cellularity) in a cancerous tissue has been assumed to correlate with the variant allele fraction (VAF) of an identified pathogenic variant. Many laboratories use the tumor cellularity as part of a quality criteria for specimen processing and clinical reporting. However, a systematic study of such correlation has yet to be shown. We performed a relatively large-scale study to determine whether pathologist-estimated tumor cellularity is correlated with next-generation sequencing (NGS)-derived VAF. **Materials and Methods:** A total of 1511 non-small cell lung cancer (NSCLC) and colorectal cancer (CRC) specimens, including formalin-fixed paraffin-embedded (FFPE) and fine needle aspirated (FNA) tissues, were analyzed by cancer hotspot NGS. For a given specimen, pathogenic variants of *BRAF*, *EGFR*, *KRAS*, and *NRAS* were identified and the determined VAFs were correlated with the corresponding tissue tumor cellularity. **Results:** The coefficient of determination R-squared (R^2^) values were calculated for each correlation. All R^2^ values were lower than 0.25, indicating poor correlations. Pathogenic variants were found, not uncommonly, in tumor specimens that carried 10% or lower tumor cellularity. There were no apparent differences of R^2^ values between the FFPE and FNA specimens. **Conclusion:** In both NSCLC and CRC, the lack of linear relationship between tumor cellularity and VAF was found across a wide range of tumor cell percentages. Caution should be used when using tumor cellularity to triage specimens for NGS testing. The tumor cellularity should be considered in relation to the limit of detection of the specific assay for the proper interpretation of a negative test result.

## 1. Introduction

Accurate mutation detection is crucial for the management of oncologic patients in the era of precision medicine [1,2]. Based on the general assumption that a gene has two copies in a human genome and a variant or mutant allele is present in every malignant cell, one would predict the number of a given mutant allele in a gene is proportionally correlated to the tumor cellularity in a cancer specimen. To measure this, the relative abundance of the mutant allele, also known as the variant allele fraction (VAF), is typically calculated by the sequence analysis pipeline using the number of mutant alleles divided by the sum of both mutant and wild-type alleles at the same mutation hotspot. In other words, VAF refers to the percentage of the variant reads among the total reads at a given genomic position. In general, one would expect that the VAF of a driver mutation correlates with the percentage of tumor content in a cancerous tissue [3,4,5,6,7]. For example, in studies of germline changes, a typical variant identified in a diploid genome can be either heterozygous or homozygous with the corresponding VAFs near 50% or 100%, respectively [8]. For the study of sporadic cancers, a cancerous tissue with 20% tumor cellularity would theoretically have 10% VAF of a given mutant allele [9].

Next-generation sequencing (NGS) has been widely used in detecting sequence changes in cancers to identify somatic variants. In contrast to the Sanger sequencing or real-time PCR methods, the VAF information from NGS tests is usually quantitative and readily available. Because the limit of detection (LoD) for the majority of NGS tests is at or around 5% VAF, low-level variants (VAF < 5%) are sometimes difficult to identify in a specimen with low tumor cellularity (<10%) [10]. Therefore, false-negative NGS test results may not be excluded when testing specimens with low tumor content. In this regard, the College of American Pathologist (CAP) has stated in its Molecular Pathology 2022 checklist (MOL.35795 and MOL.36108) that the tumor cell percentage in a tissue needs to be considered in solid tumor molecular diagnostics, for the proper interpretation of a negative test result. 

In this manuscript, we performed a relatively large-scale study to determine the correlation between the percentage of tumor cellularity estimated by certified anatomic pathologists and VAFs of well-studied driver mutations detected in key oncogenes (i.e., *BRAF*, *EGFR*, *KRAS*, and *NRAS*). All data were derived from tumor specimens of non-small cell lung cancer (NSCLC) and colorectal cancer (CRC). Our objective was to evaluate if the correlation is generally applicable to a broad range of tumor cellularity. We also wanted to assess whether there is any critical cellularity level that may be worth noting for the compliance of the CAP checklist when performing a molecular diagnostic test.

## 2. Materials and Methods

### 2.1. Sample Selection

A total of 1511 tumor specimens were used in this study. This cohort include 452 formalin-fixed, paraffin-embedded (FFPE) colorectal cancer (CRC) tissues and 1059 non-small cell lung cancer (NSCLC) cases. Among the NSCLC samples, 609 and 450 are FFPE and fine needle aspirated (FNA) specimens, respectively. These two tumor types were selected because they represent the most common sporadic solid tumors that have gone through molecular genetic testing in our hospital. Therefore, relatively large amounts of testing data are available for such a correlation study. The only difference between the two NSCLC specimen types was the sample collection method. The FFPE tissues were surgically resected specimens and the FNA tissues were collected from ultrasound-guided fine needle aspiration. According to the College of American Pathologists guidelines, these samples belong to two different tissue types, and should be validated separately in a molecular diagnostic laboratory to determine the mutation status. The mutation status of all samples was tested using the cancer hotspot next-generation sequencing (NGS) panel. The FFPE tissues had marked H&E sections for tumor cellularity determination as well as macro-dissection before extracting genomic DNA. The FNA sample tumor cellularity was determined on thin-prep slides and the entire specimen was used for the DNA extraction. The tumor cellularity determination in each case was examined by board-certified anatomic pathologists estimating the percentage nucleated cells from neoplastic versus non-neoplastic cells under a microscope. The determination may be highly variable and subjective from pathologist to pathologist. This study was performed under the approved IRB protocol (ID:17-177).

### 2.2. DNA Extraction

The DNA extraction and NGS library preparation and sequencing were described previously [9]. Briefly, DNA was isolated from FFPE (stored at room temperature) and formalin-fixed cell block tissue (stored at room temperature) utilizing the Maxwell 16 platform (Promega, Madison, WI, USA) in accordance with standard laboratory procedures. DNA was isolated from liquid cytology specimens (FNA, stored at 4 °C, collected in Cytolyt^®^ or PreservCyt^®^ preservative) utilizing the QIAsymphony^®^ SP instrument and the QIAsymphony DNA Mini kit (Qiagen, Hilden, Germany) or manually using the Gentra^®^ Puregene^®^ kit (Qiagen, Hilden, Germany) in accordance with standard laboratory procedures. DNA was quantitated and assessed for integrity using the Nanodrop 8000 spectrophotometer (ThermoFisher, Waltham, MA, USA) and the Qubit^®^ 2.0 fluorimeter (ThermoFisher, Waltham, MA, USA). A DNA OD 260/280 ratio of approximately 1.8 is generally accepted as “pure”. The extracted genomic DNA was stored at 4 °C for 1–2 months. The genomic DNA was kept in −80 °C for long-term storage. 

### 2.3. Cancer Hotspot Panel Library Preparation, Sequencing, and Data Analysis

NGS library preparation and sequencing was described previously [9]. Briefly, 10 ng of genomic DNA was recommended and used as the starting template for initial PCR amplification. AmpliSeq Cancer Hotspot Panel v2.0 PCR primers, 5X Ion AmpliSeq HiFi Mix, gDNA at 5–10 ng/µL, and molecular biology grade water were used for initial amplification according to the manufacture’s recommendation (ThermoFisher Scientific, Waltham, MA, USA). The initial PCR amplification was performed at 99 °C, 2 min for initial enzyme activation, followed by 20 cycles of 99 °C 15 s for denaturation and 60 °C 4 min for annealing and extension. The sample plate may be held at 4–10 °C overnight or frozen −20 °C for long-term storage. 

A total of 207 PCR primer pairs were used in the multiplex PCR to analyze approximately 2800 hotspot mutations in 50 genes. However, only mutation hotspots in *BRAF*, *EGFR*, *KRAS*, and *NRAS* genes were bioinformatically selected for variant annotation and interpretation. The definition of pathogenic variants is based on previous publications [11]. The sequence changes in the remaining genes were bioinformatically masked from annotation and interpretation, therefore, not even variant scientists or laboratory directors were able to view them if there are any. Short oligonucleotide sequences representing Illumina P5 and P7 flow cell capture sequences and individual library barcodes were introduced into each amplicon using the GeneRead library kit (Qiagen, Germantown, MD, USA) to accommodate the Illumina sequencer platform and properly separate the sequencing reads of individual sample libraries. Briefly, after the initial PCR amplification and the first AMPure bead cleanup, the amplicons (around 23 µL) were mixed with the 2.5 µL end repair 10X buffer and 2 µL end repair enzyme mix for each sample, then incubated at 25 °C for 30 min followed by 75 °C for 20 min. Subsequently, 3 µL A addition buffer was added and 3 µL Klenow fragment for each sample, then further incubated at 37 °C for 30 min followed by 75 °C for 10 min. In the adapter ligation step, 45 µL ligation buffer was added, 2X of 4 µL T4 DNA ligase, and 9 µL RNase-free water for each sample and incubated at 25 °C for 10 min. At the final universal amplification step, 25 µL HiFi PCR 2X master mix, 1.5 µL primer mix at 10 µM each for each sample was added. The final universal PCR amplification was performed at 98 °C, 2 min for initial enzyme activation, followed by 10 cycles of 98 °C 20 s for denaturation, 60 °C 30 s for annealing, and 72 °C 30 s for extension. After 10 PCR cycles, a final extension step was applied at 72 °C for 1 min. 

Followed by AMPure beads clean-up, the multiplex amplicon libraries were analyzed on the Bioanalyzer 2100 (Agilent Technologies, Santa Clara, CA, USA) following the manufacture’s recommendations. Amplified libraries with TruSeq adapters should have amplicon peaks in the 230–350 bp size range. For a library concentration greater than 20,000 pM, the library 1:10 with Qiagen EB Buffer was diluted and the quantitation to obtain a more accurate measurement was repeated. However, a Bioanalyzer reading of 250 pM or less following library construction was used for the indication of inadequate amplification. The no template control (NTC) must have a concentration ≤ 50 pM. If the NTC is >51 pM, the entire library preparation is subject to being repeated due to the potential contamination issue. Although the recommended input DNA amount was at 10 ng, 10 specimens with input DNA levels less than the recommended 10 ng were also tested. In total, 12 specimens of input levels ranging from 1.23 ng to 9.7 ng were also successfully sequenced and all the required sequencing quality metrics were met. Each of the 12 sequenced specimens contained either a deletion, missense variant, or a benign polymorphism. The percent of variant allele fraction, depth of coverage, and quality scores of these samples allow for confident testing of sub-optimal specimens. The concentrations of each of the prepared libraries were normalized to 2 nM then pooled at the final 4 pM concentration to sequence on a MiSeq instrument. A small amount of PhiX control (10 µL at 10 pM) was spiked into every MiSeq run. In general, pooled libraries of up to 23 clinical samples plus a positive control (HD200, multiplex FFPE reference standard, Horizon Diagnostics cat # HD200) were sequenced using Illumina standard v2, 2 × 150 bp cycle sequencing cartridge. Because the HD200 control does not carry a known deletion variant, the HD200 extracted DNA was diluted to 10 ng/µL, and further diluted 1:2 (50%) with a previously tested patient sample with a known EGFR exon 19 deletion (with at least a 20% mutant allele frequency) also diluted to 10 ng/µL. Testing of this positive control mixture was performed on every sequencing run. Sequencing was conducted according to the manufacturer’s instructions.

The sequencing data were aligned to human genome build 19 (Hg19) and hotspot mutation variants in a subset of *BRAF*, *EGFR*, *KRAS*, and *NRAS* genes were identified using NextGENe Software v. 2.4.2.3 (Soft Genetics, State College, PA, USA). The NextGENe viewer was used to visually inspect the quality of read alignment (pile-up) and variant calls, such as the presence of nucleotide homopolymer regions, the position of a variant, and the annotation of the sequencing results. The quality score Q20 was applied in filtering a given sequencing read, in which the regions of any three consecutive bases with a quality score less than Q20 were trimmed from FASTQ files along with the remainder of the downstream sequence. The average read depth for a given sample was greater than 800 reads in the targeted genomic regions. Sequencing libraries that did not deliver the minimum 100x coverage were subject to resequencing and repeating the library preparation. In general, quality scores of Q30 or higher were applied to 90–95% of the sequencing reads. Variants of 10% or lower VAFs were sequenced with a minimum read coverage of 500 reads. The cancer hotspot NGS workflow is validated to identify variants as low as 2% VAF. Single nucleotide polymorphisms with general population frequencies greater than 0.5% were not included in this study. Variants with an allele frequency of 2–20% and strand balance (forward and reverse reads) ratio less than 0.05 were also filtered from the subsequent variant annotation and interpretation. 

This study is confined to the listed hotspot regions within the tested genes shown below or, in rare cases, the regions immediately surrounding the hotspots. Gene rearrangements or copy number changes were not detected. The codon positions of mutation hotspots that were interrogated in the NGS panel were: *BRAF* (NM_004333.4: codons 444, 453, 462–471, 581–587, 592–608); *EGFR* (NM_005228.3: codons 108, 289, 596–598, 698, 702, 703, 709, 712, 719–724, 730–754, 761, 765–779, 784–786, 790–792, 810, 814, 819–821, 858–864, 868–874); *KRAS* (NM_033360.2: codons 8–22, 58–61, 117, 146); and *NRAS* (NM_002524.4: codons 11–13, 18, 60–65, 146). A small set of samples (less than five) with more than one driver mutation in each case were excluded in this study.

## 3. Results

Oncogenes *BRAF*, *EGFR*, *KRAS*, and *NRAS* are among the most mutated genes in NSCLC and CRC. Among the NSCLC cases, specimens were divided into FFPE and FNA groups while the CRC cases had only FFPE specimens for variant determination. In this study, we included only pathogenic and likely pathogenic variants identified in mutation hotspots in these four genes (Table 1, Table 2 and Table 3). In terms of the mutant frequencies in the NSCLC specimens, *EGFR* exon 19 deletions were among the most mutated variants (43.5% vs. 50.9%), followed by L858R (36.3% vs. 29.3%), and exon 20 insertions (7.7% vs. 9.5%) in both FFPE and FNA groups, respectively. Among the *KRAS* variants, the most frequent mutations occurred in the hotspots of *KRAS* codon 12 (84.9% vs. 87%), followed by codon 13 (8.9% vs. 6.3%) and codon 61 (5.2% vs. 6%), respectively, among the two specimen groups. In the *BRAF* mutation hotspots, codon 600 was the most mutated region (37.8% vs. 24.2%), followed by codon 469 (18.9% vs. 27.3%) and few other hotspot codons in both FFPE and FNA groups. The observed mutant frequencies were comparable in both tissue types and were consistent with those published in the literature [12,13,14], suggesting that no apparent selection bias was present in the recruitment of study specimens. 

For the mutations observed in the CRC FFPE specimens, *KRAS* codon 12 was the most mutated region (66.9%), followed by variants in codon 13 (18.7%) and codon 61 (5.5%). In the *BRAF* and NRAS hotspot regions, V600E (86.4%) and codon 61 (65.2%) represented the majority of mutant alleles, respectively. Like the mutation profiles seen in NSCLC, the observed mutation frequencies in CRC were similar to those previously published [15,16,17], which further demonstrates the CRC specimens in this study are somewhat randomly selected without bias. It is worth noting that the hotspot mutations shown in this study are mutually exclusive in general, therefore, each tissue specimen corresponds to a single variant or one VAF. The specimens with more than one variant identified, represented less than an estimated 0.3% in the total study cohort, are excluded from this study. 

**Figure 1:**
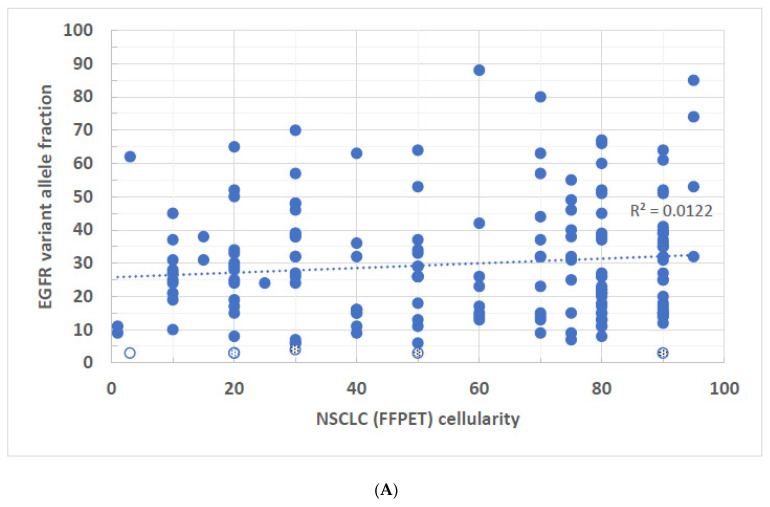

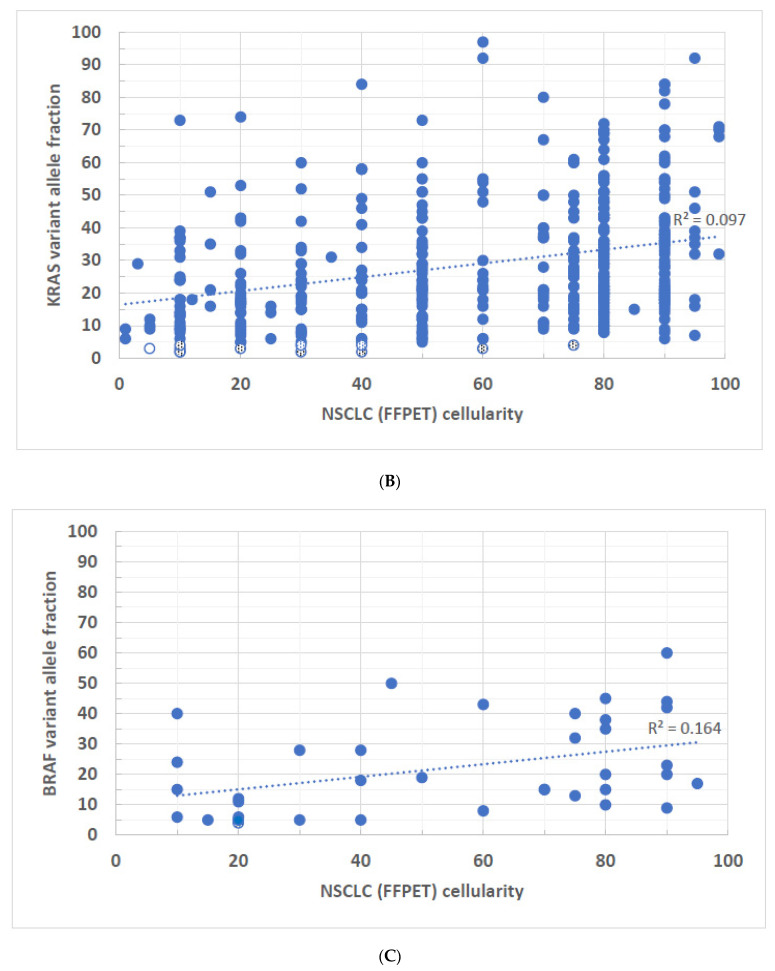
(**A**–**C**)**:** VAFs of disease-associated variants derived from FFPE specimens of NSCLC were plotted against the tumor cellularity of the same tumor tissue. (**A**) *EGFR* variants, R^2^ = 0.012, *p* = 0.153. (**B**) *KRAS* variants, R^2^ = 0.097, *p* < 0.001. (**C**) *BRAF* variants R^2^ = 0.164, *p* < 0.013. Open circles represent samples of less than 10% tumor cellularity as well as identified VAFs at equal or less than 5%. Dotted circles represent samples of equal or greater than 10% tumor cellularity but have identified VAFs at equal or less than 5%.

**Figure 2:**
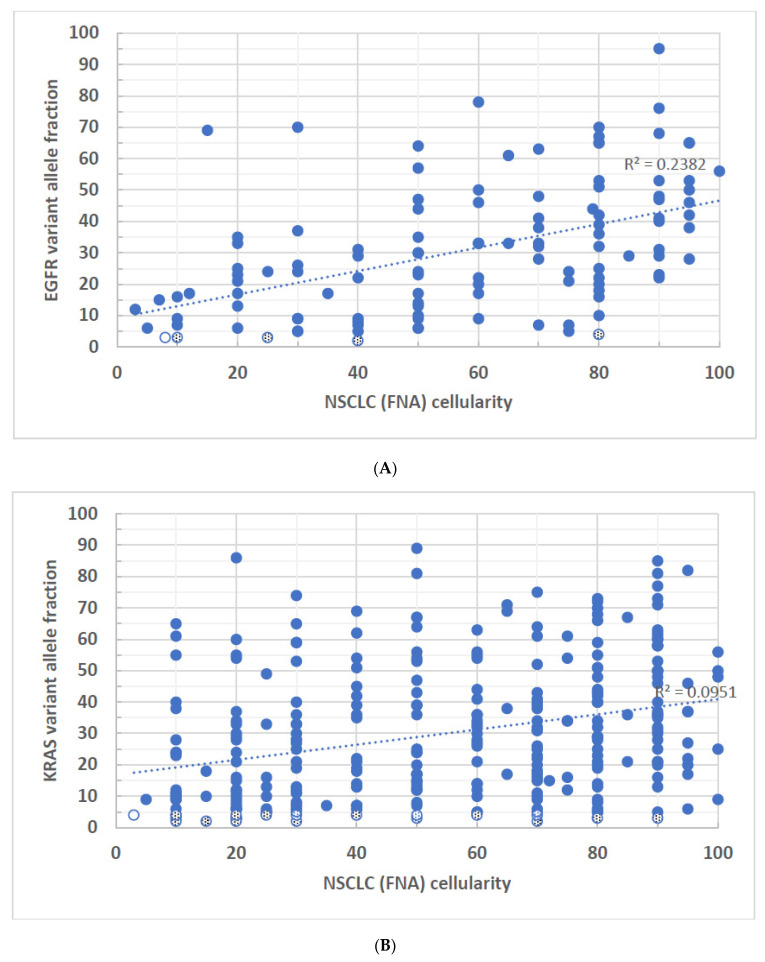

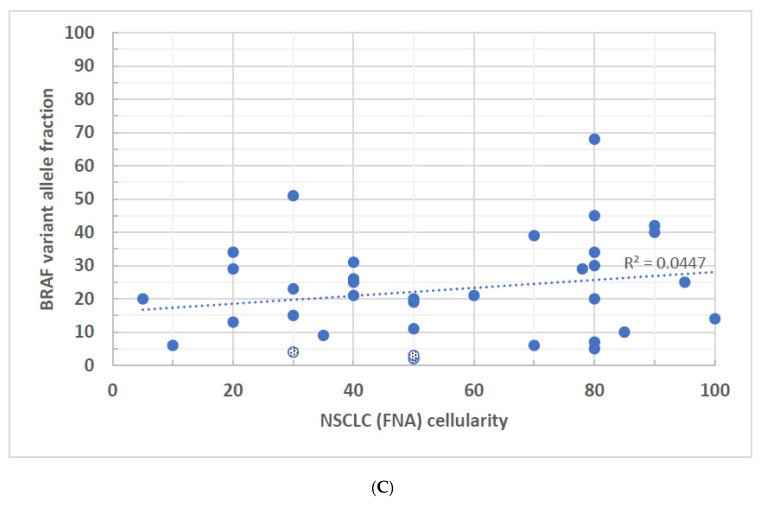
(**A**–**C**)**:** VAFs of disease-associated variants derived from FNA specimens of NSCLC were plotted against the tumor cellularity of the same tumor tissue. (**A**) *EGFR* variants, R^2^ = 0.238, *p* < 0.001. (**B**) *KRAS* variants, R^2^ = 0.095, *p* < 0.001. (**C**) *BRAF* variants, R^2^ = 0.044, *p* = 0.223. Open circles represent samples of less than 10% tumor cellularity as well as identified VAFs at equal or less than 5%. Dotted circles represent samples of equal or greater than 10% tumor cellularity but have identified VAFs at equal or less than 5%.

**Figure 3:**
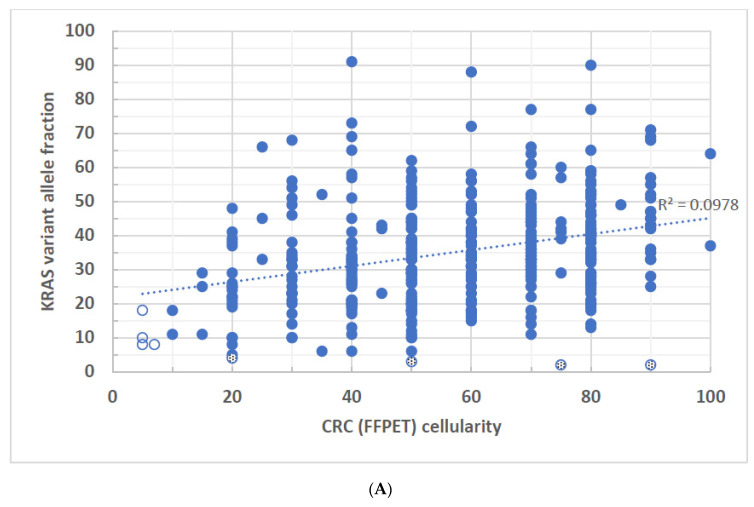

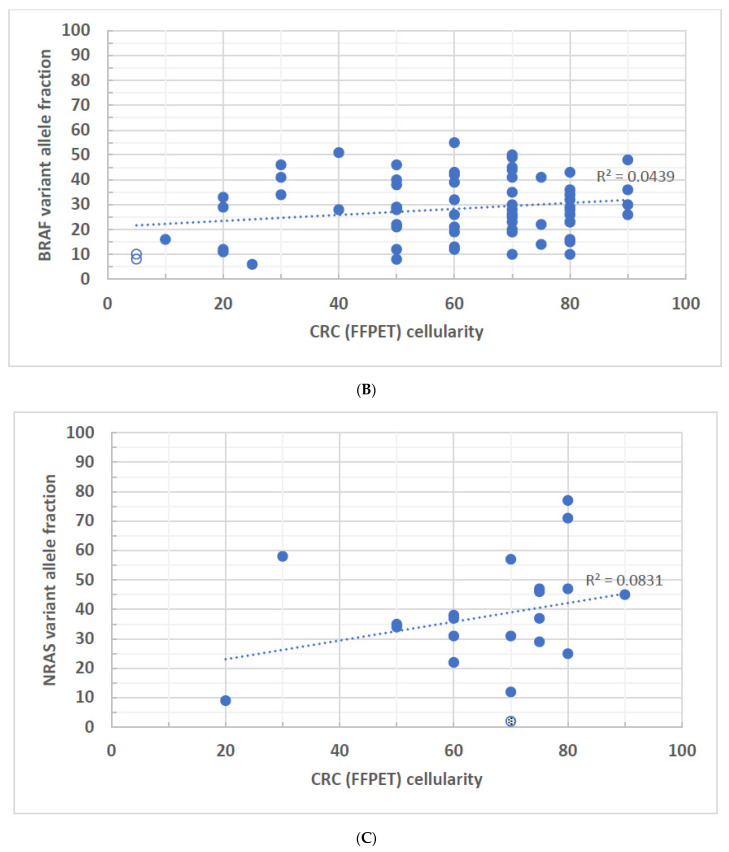
(**A**–**C**): VAFs of disease-associated variants derived from FFPE specimens of CRC were plotted against the tumor cellularity of the same tumor tissue. (**A**) *KRAS* variants, R^2^ = 0.097, *p* < 0.001. (**B**) *BRAF* variants, R^2^ = 0.044, *p* = 0.091. (**C**) *NRAS* variants, R^2^ = 0.083, *p* = 0.205. Open circles represent samples of less than 10% tumor cellularity but have identified VAFs at equal or greater than 5%. Dotted circles represent samples of equal or greater than 10% tumor cellularity but have identified VAFs at equal or less than 5%.

To determine the correlation between mutant VAF and the tumor cell percentage, a basic regression model was applied to each correlation, and the coefficient of determination R-squared (R^2^) values for each gene are shown. R^2^ measures the extent of the variance of one variable correlated to the variance of the second variable. For example, if the R^2^ is 0.5, it suggests that approximately half of the observed variation may be explained by the model. In the NSCLC cases, *EGFR*-FFPE and *BRAF*-FNA specimens showed no statistically significant correlations between VAF and tumor cellularity (Figure 1A and Figure 2C). Even though the statistically significant correlations were seen in *KRAS*-FFPE, *BRAF*-FFPE, *EGFR*-FNA, and *KRAS*-FNA samples, with the highest R^2^ value 0.238 found in *EGFR* FNA specimens (Figure 2A), these data suggest that in the best-case scenario, only 23.8% of *EGFR* VAF samples may be correlated with tumor cellularity. In other words, at least 76.2% of VAFs were not explained by tumor cellularity. Also, for a given gene, there were no apparent differences of R^2^ values between FFPE and FNA specimens. Although trends of higher tumor cellularity associated with higher VAFs were seen in *EGFR* variants from the NSCLC-FNA specimens (Figure 2A) and *KRAS* variants from the CRC-FFPE specimens (Figure 3A), the general observation shows a poor or even no correlation between the VAF and specimen’s tumor cellularity regardless of the gene origins or specimen types. There were 16 FFPE (~2.6%, n = 609) and 25 FNA (~5.6%, n = 450) NSCLC samples that had disease-associated variants with <5% VAFs (Figure 1A–C and Figure 2A–C shown in both dotted and open circles), which may not be identified by molecular methods that have LoD > 5%. Notice that four of these low-level variants were derived from samples with tumor cellularity less than 10% (Figure 1A,B and Figure 2A,B shown in open circles). All these low-level variants are present in a wide range (3–90%) of tumor cellularity.

Similar low R^2^ values were also seen in the CRC cases (Figure 3A–C) where the FFPE tissue was the only sample source. Only 9.7% of *KRAS* VAFs (R^2^ = 0.097) may be statistically significantly correlated with tumor cellularity (Figure 3A). Neither *BRAF* nor *NRAS* showed any statistically significant correlation between VAF and tumor cellularity (Figure 3B,C). All the data suggest a lack of linear relationship between tumor cell percentage and mutant VAFs in *BRAF, KRAS*, and *NRAS* in CRC. Although there were 6 FFPE (~1.3%, n = 452) CRC specimens with <10% tumor cellularity, all the variants identified in these samples had VAFs > 5% (Figure 3A,B, open circles). Notice that 5 of the CRC specimens had 20% or greater tumor cellularity but the identified VAFs were less than 5% (Figure 3A,C, dotted circles), including a specimen of high tumor cellularity (70%) but low VAF variant (<10%) observed in *NRAS* (Figure 3C, a dotted circle). *NRAS* variants did not present as often as *BRAF* and *KRAS* variants in CRC.

We observed, in both NSCLC and CRC, the poor or no correlation between VAF and tumor cellularity in a wide range of tumor cell percentages. Low VAF (<5%) can be found in specimens with any level of tumor cells (10–90%). In general, specimens of low tumor cell percentage were linked to low VAFs, for example, samples with 20% or lower tumor cellularity often show variants with lower than 50% VAFs. However, specimens of high tumor cellularity (80% or higher) were not necessarily linked to higher VAFs, instead, 20% or lower VAFs were not uncommonly seen.

## 4. Discussion

The discordance between VAFs and pathologist-determined tumor cellularity may be attributed to the following reasons. First, although the assessment of tumor cellularity in surgically removed tissues was performed by board-certified pathologists, it may not be as accurate as one would expect. The intertwined cancerous and non-cancerous cells in a three-dimensional tissue context makes it extremely challenging for an accurate estimation of tumor cellularity under a microscope. Furthermore, this human estimation bias may vary from pathologist to pathologist. When comparing tumor cellularity estimated by pathologists to a standard counting software, Smiths et al. found that one-third of them were overestimated, while 17% of cases were underestimated in the same study [18]. Our results suggest that a more reliable procedure to generate a consistent tumor cellularity estimation, such as automated image analysis or machine learning technology coupled with statistical modelings, may be evaluated to minimize unintentional human subjective bias in the estimation procedure [19,20].

Second, tumor cellularity describes the amount of morphologically abnormal cells in a tissue. However, it does not necessarily explain the abnormal gene sequence changes at the molecular level. Thus, the general assumption that disease-associated variants present merely in morphologically abnormal cells may not be appropriate for the correlation of VAF and tumor cellularity. Several studies have shown that the histologically normal-appearing cells carry disease-associated somatic variants [21,22,23]. The genetic abnormalities, particularly those in adjacent “normal appearing” cells are not visible by routine histological examination. Therefore, the tumor cellularity determined by trained pathologists may be an under-estimation of the true genetic defects. The skewed correlation results may be attributed to the disease-associated variants in the “normal-appearing” cells that were not counted in the general assumption. 

Third, the supposed linear correlation between tumor cellularity and VAF was based on the assumption that the diploid human genome is present in every cell in cancerous tissue. Nevertheless, many tumor cells have genomic rearrangement where portions of the chromosomes have undergone copy number alternations (e.g., gains or losses) resulting in an aneuploid genome [24]. Thus, one can no longer assume each tumor cell contributes one or some defined copies of the mutant allele(s) without knowing the copy number information of the tumor. In reality, because a tumor cell most likely no longer retains its diploid genome status, the VAFs of somatic variants can theoretically range from 0 to 100% depending on the copy number of a mutant allele and the amount of affected cells in a malignant tissue. 

Fourth, although sporadic cancer is expected to originate from a single cell, as it often becomes quite heterogeneous morphologically at the time of diagnosis [25]. This tumor heterogeneity is likely attributed to monoclonal or multiclonal expansions during tumor progression. Driver mutations may be amplified by clonal expansion or may only present in one of these clones followed by secondary genetic alterations, which partially explains why the mutant VAF may not come in proportion with the percentage of morphologically abnormal cells in a tumor tissue. Therefore, clonality is one of the reasons that may explain the lack of correlation between tumor cellularity and VAF of the pathogenic variants.

It is standard practice in a molecular diagnostic laboratory to report the percentage of neoplastic cell content for an FFPE tumor specimen if a molecular genetic diagnosis is needed. This is because one typically assumes the tumor cellularity level would correlate with the VAF. When a heterogeneous low tumor content sample was tested and the negative test result was shown, the possibility of false-negative or missing low VAF variants may not be completely excluded. Therefore, some laboratories may use the tumor cellularity as a cut-off to determine which specimens should be analyzed or not. However, we have shown that simply applying tumor cellularity cut-off would have omitted specimens with identifiable pathogenic variants, especially when the test LoD was not considered. 

It is worth noting that pathogenic variants were identified in a total of 92 (85 NSCLC and 7 CRC) cases with 10% or lower tumor cellularity. Even at 5% or lower tumor cellularity cut-off, a total of 21 (16 NSCLC and 5 CRC) cases were shown to contain pathogenic variants. If these samples had been triaged and cancelled for NGS testing based on the cut-off of 5% or higher tumor cellularity, these patients (~1.5% NSCLC and ~1.1% CRC at 5% cut-off, or ~8% NSCLC and ~1.5% CRC at 10% cut-off) would have missed the invaluable opportunity for identification of actionable pathogenic variants. 

In theory, the LoD of a test becomes important only when a tissue sample carries a low amount of tumor cells. This is because the low-level mutant alleles may be difficult to identify in a mixture of low tumor and high normal cell population. However, our data have shown that low VAF may exist in specimens with a wide range of tumor cellularity for the reasons discussed above, including those with high tumor content. In this regard, LoD of a clinical test becomes an essential factor regardless of the tumor cellularity in a tissue when interpreting negative results. Since our cancer hotspot test is able to detect a variant as low as 2% VAF, our routine clinical practice processes every tumor specimen irrespective of the tumor cell percentages. Consequently, depending on the LoD of a molecular diagnostic method, the consideration of false-negative test results should not merely rely on the percentage of tumor cells of a specimen. In summary, our data show the importance of analyzing tumor cell percentage as well as understanding the LoD of a molecular diagnostic test, as the CAP checklist indicated, to properly interpret the sequencing results. Using only tumor cellularity to triage tumor specimens for NGS test may unnecessarily exclude invaluable opportunities for critical patient care.

## Figures and Tables

**Table 1:** The variants found in the NSCLC FFPE specimens.

Gene	Variant	Number	Percentage
EGFR	Ex19 deletion	73	43.5%
	Ex19 insertion	3	1.8%
	Ex20 insertion	13	7.7%
	G719A/S	4	2.4%
	L747P	2	1.2%
	L858R	61	36.3%
	L861Q	4	2.4%
	M600V	2	1.2%
	Other (P733Q, R776C, T751I, V742I, V769L)	6	3.6%
KRAS	G12A/C/D/F/R/S/V	343	84.9%
	G13C/D/E/F/R/V	36	8.9%
	L19F	2	0.5%
	A59G/T	2	0.5%
	Q61H/L/R	21	5.2%
BRAF	G464V	2	5.4%
	G466R/V	3	8.1%
	G469A/E/R/S/V	7	18.9%
	N581I/S	5	13.5%
	D594G/N	3	8.1%
	V600E/K	14	37.8%
	Other (L597R, R462T, p.Thr599_Val600delinsMet)	3	8.1%

**Table 2:** The variants found in the NSCLC FNA specimens.

Gene	Variant	Number	Percentage
EGFR	Ex19 deletion	59	50.9%
	Ex19 insertion	1	0.9%
	Ex20 insertion	11	9.5%
	G719A/S	4	3.4%
	L858R	34	29.3%
	L861Q	4	3.4%
	Other (G721A, L747P, R776L)	3	2.6%
KRAS	G12A/C/D/F/R/S/V	262	87.0%
	G13C/D/R/V	19	6.3%
	Q61H/K/L	18	6.0%
	A146T/V	2	0.7%
BRAF	G464V	1	3.0%
	G466A/V	5	15.2%
	G469A/R/V	9	27.3%
	N581I/S	3	9.1%
	N594G/N	5	15.2%
	K601E	2	6.1%
	V600E	8	24.2%

**Table 3:** The variants found in the CRC FFPE specimens.

Gene	Variant	Number	Percentage
KRAS	G12A/C/D/F/R/S/V	243	66.9%
	G13C/D/R	68	18.7%
	Q22K	2	0.6%
	A59T	2	0.6%
	Q61H/K/L/R	20	5.5%
	K117N	3	0.8%
	A146R	1	0.3%
	A146T	24	6.6%
BRAF	D594G	3	4.5%
	V600E	57	86.4%
	Other (G466R, F468L, G469A, D581I, G596R, K601N)	6	9.1%
NRAS	G12A/C/D/S	6	26.1%
	G13R	2	8.7%
	Q61K/L/R	15	65.2%

## Data Availability

The datasets presented in this article are not readily available because privacy, legal or ethical reasons. Requests to access the datasets should be directed to the corresponding author.
